# Pasteurism

**Published:** 1886-12

**Authors:** 


### EDITORIAL.

PASTEURISM.

At a recent meeting of the Academy of Sciences, in Paris, M. Pasteur presented in a communication to that body his latest conclusions as to the methods and results of anti-rabic inoculations. During the year ending November 1st, 1886, only three persons died in the hospitals of Paris from hydrophobia. Of these two had not been treated by Pasteur's method, and the third had been subjected to a form of treatment since abandoned as insufficient. During the previous five years sixty persons had died in the hospitals, and during the year ending November 1st, 1885, thirty-one had died. In all, 2,190 persons were treated during the year. In France and Algeria, 1,700, of whom ten died. It is not certain that every one of these persons was bitten by a rabid animal, but as M. Pasteur very justly remarks, it is almost certain that the larger portion of individuals bitten were subjected to treatment, and yet there were known to have been seventeen deaths from hydrophobia in France and Algeria in addition to the ten of the 1,700 cases treated. Taken together, these facts seem to demonstrate the efficacy of the treatment. Grave doubts have been entertained as to the safety of inoculations such as Pasteur has practiced. It has been thought that the introduction into the human organism of an emulsion of the spinal cord of an animal, dead from such a disease, in the light of what we know of infection and septic processes, must be dangerous. In answer to such an objection Pasteur points to his more than 2,000 cases in a single year, with not a single accident. The failures he attributes to the fact that the process has been too slow. When the wounds are upon the face, the most dangerous location, or are deep upon other parts of the body, he proceeds to repeat the inoculations, commencing with cords of twelve days, say at 1 P.M., of ten days at 4 P.M., and of eight days at 9 P.M. On the next day he operates with cords of six, of four and of two days at the same hours. The third day he inoculates with the cord of an animal dead only one day, that is with a practically fresh cord. This process is repeated, commencing with a cord of eight days, and coming down to the fresh cord again, and in this way, to use his own words, "there are made three treatments in ten days, carrying each one to the fresh cord." He believes some, at least, of his failures have been due to the fact that the inoculations have not been made with sufficient frequency, or, in other words, the fresh virus has not been reached early enough.

There seems to be no longer room for doubt as to the fact that this process does prevent the development of rabies in the human subject bitten by rabid animals. Animals inoculated with the rabic virus are also protected by *Pasteurism*. The explanation of this fact, however, is still to be sought. Neither chemistry nor the microscope has revealed the morbific agent or thrown light upon what seems to be a curious fact, namely, that the repetition of the virus produces an arrest of the incubating process. This is not homœopathic, but idiopathic. It would seem that the poet's lines,

> "Shallow draughts intoxicate the brain,
> Drinking largely sobers us again,"

literally becomes true.

